# Suppressing miR-199a-3p by promoter methylation contributes to tumor aggressiveness and cisplatin resistance of ovarian cancer through promoting DDR1 expression

**DOI:** 10.1186/s13048-017-0333-4

**Published:** 2017-07-25

**Authors:** Yuao Deng, Fang Zhao, Liu Hui, Xiuyun Li, Danyu Zhang, Wang Lin, Zhiqiang Chen, Yingxia Ning

**Affiliations:** 1 Department of Gynecology and Obstetrics, Shenzhen People’s Hospital, Shenzhen, 518020 China; 2 Department of Infectious Disease, The third people’s Hospital of Shenzhen, Shenzhen, 518112 China; 3 Department of Gynecology and Obstetrics, The Twelfth People’s Hospital of Guangzho, Guangzhou, 510120 China; 4 Department of Pathology, Shenzhen People’s Hospital, Shenzhen, 518020 China; 5 Department of Gynecology and Obstetrics, The First Affiliated Hospital of Guangzhou Medical University, Guangzhou, 510120 China

**Keywords:** Ovarian cancer, miR-199a-3p, Discoidin domain receptor 1 (DDR1), Cisplatin

## Abstract

**Background:**

Discoidin Domain Receptor 1 (DDR1) belongs to the family of collagen receptor tyrosine kinases that confers the progression of various cancers. Aberrant expression of DDR1 was detected in several human cancers including ovarian cancer, which had been shown to increase the migration and invasion of tumor cells. However, the precise mechanisms underlying the abnormal expression of DDR1 in ovarian cancer has not been well investigated in previous studies.

**Results:**

In this work, a negative correlation between DDR1 and a tumor suppressor miRNA, miR-199a-3p, was observed in ovarian cancer tissues. Furthermore, in vitro experimental results confirmed that miR-199a-3p decreased the expression of DDR1 via targeting the 3’UTR of DDR1 mRNA. To explore the mechanisms for miR-199a-3p silence in ovarian cancer, the methylation status of the miR-199a promoter was analyzed in ovarian epithelial or cancer cells by methylation-specific PCR and bisulphite sequencing. As expected, the miR-199a promoter was hypermethylated in ovarian cancer cells but not in normal ovarianepithelial cells. Interestingly, knockdown of DNA methyltransferase 3A (DNMT3A) notably increased miR-199a-3p level and then attenuated the expression of DDR1 in ovarian cancer cells, which suggested that DNMT3A was responsible for the miR-199a promoter hypermethylation. Phenotype experiments showed that overexpression of miR-199a-3p significantly impaired the migratory, invasive, and tumorigenic capabilities of ovarian cancer cells as well as enhanced cisplatin resistance through inhibiting DDR1 expression.

**Conclusion:**

These findings demonstrate a critical role of miR-199a-3p/DDR1 pathway in ovarian cancer development.

## Background

As one of the most common gynecological malignant tumors, ovarian cancer has become a serious problem for female healthy because of its high morbidity and mortality. However, more than 75% of women present with locally advanced or disseminated ovarian cancer at the time of diagnosis [1]. Even though standard therapies including cisplatin-based combination chemotherapy and cytoreductive surgery have been widely applied in the clinical treatment of advanced-stage ovarian cancer, the overall 5-year survival rate for metastatic ovarian cancer is only 15–30% [2, 3]. As a main cause, development of resistance to platinum results in the failure of chemotherapy during the treatment of ovarian cancer [4]. Unfortunately, the mechanisms of platinum resistance have not been clearly demonstrated in ovarian cancer. In the present study, deregulation of Discoidin Domain Receptor 1 (DDR1) in ovarian cancer was found to enhance the resistance to cisplatin.

The discoidin domain receptor, DDR1, belongs to the family of receptor tyrosine kinases (RTKs) which transmit signals into cells by providing docking sites for effector molecules in the form of phosphorylated cytoplasmic tyrosines [5]. DDRs regulate important aspects of cellular processes including proliferation, migration, adhesion, and ECM remodeling by interacting with several types of collagen [6]. In several human cancers, such as breast cancer, lung cancer, and hepatocarcinoma, dysregulated DDRs have been observed to be related with tumor development [7]. Like many other RTKs, the DDRs display a critical role in cancer progression, in part by modulating the interaction of cancer cells with collagens [7, 8]. Moreover, DDR1 was reported to decrease the sensitivity to chemotherapy and induce survival signals, which may lead to neoplasm recurrence [9, 10]. However, the molecular mechanism underlying the abnormal expression of DDR1 in ovarian cancer was not well investigated in the previous studies. Below, we discovered DDR1 as a target of miR-199a-3p, a tumor-suppressor miRNA, which was silenced by hypermethylation in ovarian cancer.

As a class of small noncoding RNAs, miRNAs target the 3’UTRs of multiple mRNAs and block translational process, which leads to gene silence. One of the gene clusters targeted by miRNAs has been proved to be associated with tumor aggressiveness [11]. Mature human miR-199a contains two derivatives: miR-199a-5p and miR-199a-3p. The same pre-miR-199a is encoded by two loci in the human genome, miR-199a-1 in chromosome 19 and miR-199a-2 in chromosome 1. miR-199a-3p functions as a tumor suppressor and inhibits tumor metastasis by repressing various oncogenes, such as mTOR,aurora kinase A, and c-MET [12–14].Previous analysis have indicated that miR-199a-3p expression is inhibited in various cancer types [15, 16]. The low expression of miR-199a-3p has been previously detected in ovarian carcinoma and is significantly correlated with a poor prognosis [17].

In this study, miR-199a-3p is revealed as an upstream regulator of DDR1 which confers the malignance and cisplatin resistance of ovarian cancer. Ectopic low levels of miR-199a-3p accompanied with increased DDR1 expressions are detected in ovarian cancer cells or tissues. Further analysis suggest that downregulation of miR-199a-3p in ovarian cancer is resulted from the promoter hypermethylation of miR-199a gene. Therefore, our study indicates that aberrant methylation status in ovarian cancer breaks the inhibitory effect of miR-199a-3p on DDR1 expression and consequently promotes the malignant phenotypes of ovarian cancer.

## Methods

### Cell culture and reagents

Human ovarian cancer cell lines SKOV3 and HO-8910, and immortalized ovarian epithelial cell line IOSE386 were purchased from ATCC (USA) and cultured in RPMI 1640 medium (Corning, USA) supplemented with 10% fetal bovine serum at 37 °C with 5% CO2. Cisplatin and 5-Aza-dC were purchased from Sigma (USA).

### Immunohistochemistry

The study has been performed with the approval of the Ethics Committee of the Shenzhen People’s Hospital. Ovarian epithelial cancer tissues were collected by needle biopsy or surgery from 115 patients at Shenzhen People’s Hospital (Shenzhen, China), the First Affiliated Hospital of Guangzhou Medical University (Guangzhou, China), and the Twelfth People’s Hospital of Guangzhou (Guangzhou, China) from 2009 to 2012. All specimens were fixed in 10% neutral formalin, embedded in paraffin and cut into 4-μm sections for immunohistochemical staining. The EnVision™ two-step method was used (DAKO, Hamburg, Germany), as well as the following antibody: antibody against DDR1. To estimate the score for each slide, at least 10 individual fields at 200× were chosen, and 100 cancer cells were counted in each field. The immunostaining intensity was divided into four grades: 0, no expression; 1, mildly positive; 2, moderately positive; and 3, markedly positive. The proportion of positive-staining cells was divided into five grades: 0, <10%; 1, 11–25%; 2, 26–50%; 3,51–75%; and 4, >75%. The staining results were assessed and confirmed by two independent investigators blinded to the clinical data. The percentage of positivity of the tumor cells and the staining intensities were then multiplied in order to generate the IHC score, and graded as 0–6, low expression; 7–12, high expression. Cases with a discrepancy in scores were discussed to obtain a consensus.

### RNA extraction and quantitative PCR

Total RNA was isolated from clinical tissue samples with a total RNA isolation kit (AP-MN-MS-RNA, Axygen, CA, USA) as described by the manufacturer. Total RNA from each cell was isolated with Trizol Reagent (Invitrogen, NY, USA) and miRNAs were isolated with microRNA Purification Kit (Norgen Biotek, Thorold Ontario, Canada), according to the manufacturer’s protocol. miRNA-specific quantitative PCR was performed with Taqman MicroRNA Assay primers (Applied Biosystems) according to the manufacturer’s instructions. The miRNA level was normalized by U6 RNA. The qPCR for mRNA was performed with SYBR Green PCR Master Mix (Applied Biosystems).

### DNA methylation analysis

#### Methylation specific PCR (MSP)

The bisulfite conversion of genome DNA was conducted with EZ DNA Methylation-Gold Kit (Zymo Research Corporation,USA) according to the manufacturer’s instructions. The bisulfite-treated DNA was amplified with the methylation-specific primers (Forward: TCGGTCGTAATGAGAAATAGTC; Reverse: TAAACGACTCGTCGAAACA) or the unmethylated specific primers (Forward: GTTGGTTGTA ATGAGAAATAGTT; Reverse: ATAAACAACTCATCAAAACA), and the amplified products were analyzed on agarose gel and quantified by qPCR.

#### DNA sodium bisulfite conversion (BSP)

Specific primers for converted promoter region were used to generate PCR product (Forward: TATATTTGGAATTGTTTATAGT; Reverse: AAAAAAATATCTAACTCTTTAA). PCR products were cloned in pGEMTeasy (Promega) followed by sequencing with Sp6 primer.

### Chromatin immunoprecipitation assay

Chromatin was cross-linked with 1% formaldehyde and sonicated to obtain a DNA fragment of 200–500 bp. After centrifugation, the supernatants were subjected to immunoprecipitation overnight at 4 °C with antibodies against DNMT3A or normal IgG. The DNA-protein complexes were isolated using Protein A/G PLUS-Agarose (Santa Cruz). The crosslinking was reversed and released DNA fragments were purified and quantified by QPCR using the following primer pair for miR-199a promoter (Forward:CCACATCTGGAACTGTTTAC; Reverse:AATGTCTGACTCTTTGGGGG). Three independent experiments were performed.

### Wound healing assay

The indicated cells were pre-seeded in a 6-well plate. Cell monolayers were then gently scratched with a pipette tip across the entire diameter of the well. The area of denuded surface was quantified immediately after wounding and again 24 h later. Images of the wound area were captured in 6 fields using an inverted microscope.

### Invasion assay

The ability of cells to invade through matrigel was measured with Boyden’s chamber technique as previously described. The invaded cells on the filters were stained with crystal violet and then counted under the microscope. A total of five fields were counted for each transwell filter.

### Colony formation in soft agar

For colony formation experiment, cells were suspended in 0.3% agar and seeded into 6-well plate pre-coated with 1.0 ml of 0.6% agar. Medium was changed every 4 days for 3 weeks. The number and size of colonies were examined and data were obtained by analyzing with Image J software.

### Cell cytotoxicity assay

The indicated cells were seeded in 96-wellplates and cultured for 24 h followed by cisplatin treatment at different concentrations for 24 h. Cell viability was evaluated by CCK-8 assay (Kumamoto, Japan) according to the manufacturer’s instructions.

### Flow cytometric detection of apoptosis

The indicated cells were collected for detection of apoptosis using the Annexin-V-FLUOS Kit (Roche*,* USA) according to the manufacturer’s instructions. All of the samples were assayed in triplicate.

### Statistical analysis

All statistical analyses were carried out using the SPSS 17.0 statistical software. Experimental data were presented as mean ± SD from at least three independent experiments. The data were analyzed by the 2-tailed Student’s *t-*test andPearson’scorrelation test. A *p* value < 0.05 was considered statistically significant.

## Results

### A negative correlation between DDR1 and miR-199a-3p in clinical ovarian cancer tissues

**Fig. 1:**
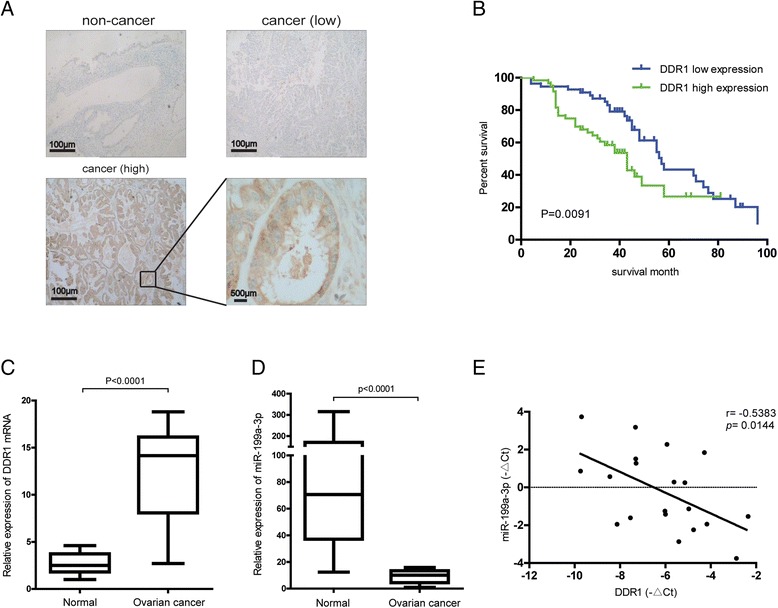
A negative correlation between DDR1 and miR-199a-3p was observed in clinical ovarian cancer tissues. **a** The representative results of DDR1 expression in human ovarian cancer tissue compared to normal, by immunohistochemical analysis. **b** Kaplan–Meier survival curves for the ovarian cancer patients divided by the expression of DDR1. **c** Relative mRNA levels of DDR1 in ovarian cancer tissues compared with normal ovarian tissues. *P < 0.05. **d** Relative expression of miR-199a-3p in ovarian cancer tissues compared with normal ovarian tissues. *P < 0.05. **e** miR-199a-3p and DDR1 mRNA expression levels inversely correlated in 20 clinical samples. Statistical analysis to evaluate correlation was performed using Pearson’s correlation analysis (*r* = −0.5383, *P* = 0.0144)

Consistent with the upregulation of DDR1 in other cancer types, both the mRNA and protein of DDR1 were aberrantly overexpressed in ovarian cancer tissues compared with the normal control (Fig. 1a and c). Further clinical analysis showed that high expression of DDR1 was statistically associated with lymph node metastasis, TNM stage and distant metastasis (*P* < 0.05) but not with age and differentiation (*P* > 0.05) (Table 1). And the 3-year overall survival (OS) rate of the group with DDR1 high expression was significantly lower than that of the group with DDR1 low expression (41.67% vs 74.55%, *P* = 0.0091) (Fig. 1b). Contrast with DDR1, miR-199a-3p was dramatically downregulated in ovarian cancer tissues (Fig. 1d). Importantly, an inverse correlation between DDR1 and miR-199a-3p was found (Fig. 1e). These data show a poor prognosis of the ovarian cancer patients with high DDR1 expression and a negative correlation between DDR1and miR-199a-3p in ovarian cancer.

**Table 1:** Correlation of the expression of DDR1 with clinicopathological features in ovarian cancer tissues

		Total	DDR1		
			low	high	*p*
		115	55	60	
Age					0.4323
	≤ 65	53	30	23	
	>65	62	25	37	
Differentiation					0.0883
	Well differentiation	35	17	18	
	Moderate differentiation	49	23	26	
	Poorly differentiation	26	13	13	
	Undifferentiation	5	2	3	
Lymph node metastasis					0.0211*
	No	61	40	21	
	Yes	54	15	39	
TNM stage					0.0073*
	I	39	23	16	
	II	31	17	14	
	III	24	11	13	
	IV	21	4	17	
Distant metastasis					0.0002*
	M0	90	49	41	
	M1	25	6	19	

* *p* value < 0.05 was considered statistically significant

### miR-199a-3p reduces DDR1 expression via targeting the 3’UTR of DDR1 mRNA in ovarian cancer cells

**Fig. 2:**
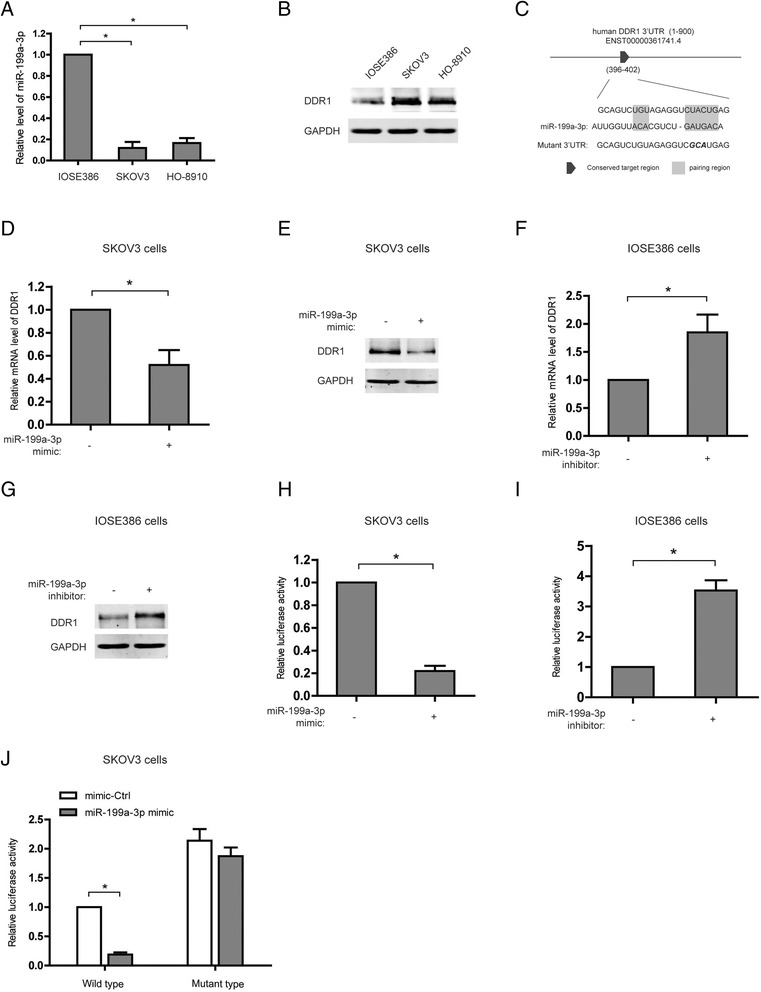
The mRNA of DDR1 is a target of miR-199a-3p in ovarian cancer cells. **a** and **b** The expression levels of miR-199a-3p (**a**) and DDR1 (**b**) in two ovarian cancer cell lines, SKOV3 and HO-8910, and a normal ovarian cell line, IOSE386. *P < 0.05. **c** The putative human DDR1–3’UTR fragment containing a wild or mutant miR-199a-3p binding site was insert into the luciferase report vector pmirGLO downstream. **d** and **e** The DDR1 mRNA (**d**)and protein (**e**) levels after the transfection with miR-199a-3p mimics or negative control in SKOV3 cells. *P < 0.05. **f** and **g** The DDR1 mRNA (**f**)and protein (**g**) levels after the transfection with an anti-miRNA inhibitor specific for miR-199a-3p or negative control in IOSE386 cells. *P < 0.05. **h** and **i**Luciferase activity of DDR1 promoter after the indicated transfection in SKOV3 (**h**) or IOSE386 (**i**) cells. *P < 0.05. **j** Luciferase assay in the SKOV3 cells co-transfected with DDR1–3’UTRconstructs containing a wild or mutantmiR-199a-3p binding site as well as miR-199a-3p mimics or scrambled oligonucleotides as the negative control. *P < 0.05

To examine the relationship between miR-199a-3p and DDR1 in ovarian cancer, the expression levels of miR-199a-3p and DDR1 were measured in two ovarian cancer cell lines, SKOV3 and HO-8910, and a normal ovarian cell line, IOSE386. Compared with IOSE386, higher expression of DDR1 accompanied with lower miR-199a-3p level was detected in SKOV3 and HO-8910 cells (Fig. 2a and b). Furthermore, a miR-199a-3p binding site located at the 3’UTR of DDR1 mRNA was predicted using the software “TargetScanHuman 7.1” (Fig. 2c). Consistently, transfection of miR-199a-3p mimic was shown to decrease the expression of DDR1 at both mRNA and protein level in SKOV3 cells (Fig. 2d and e). In addition, miR-199a-3p inhibitor displayed a promotive effect on the mRNA and protein level of DDR1 in IOSE386 cells (Fig. f and g). More importantly, the luciferase reporter vector of DDR1 3’UTR containing a wild miR-199a-3pbinding site or a corresponding mutant site was constructed to experimentally validate the target prediction (Fig. 2c). Transfection of miR-199a-3p mimic greatly attenuated the luciferase activity of DDR13’UTR reporter in SKOV3 cells; while miR-199a-3p inhibitor strengthened the luciferase activity in IOSE386 cells (Fig. 2h and i). As expected, mutation in the miR-199a-3p binding site evidently weakened the response of luciferase activity to miR-199a-3p mimic (Fig. 2j). Taken together, miR-199a-3p decreases the expression of DDR1 by directly targeting the 3’UTR of DDR1 mRNA in ovarian cancer cells.

### Promoter hypermethylation leads to downregulation of miR-199a-3p in ovarian cancer

**Fig. 3:**
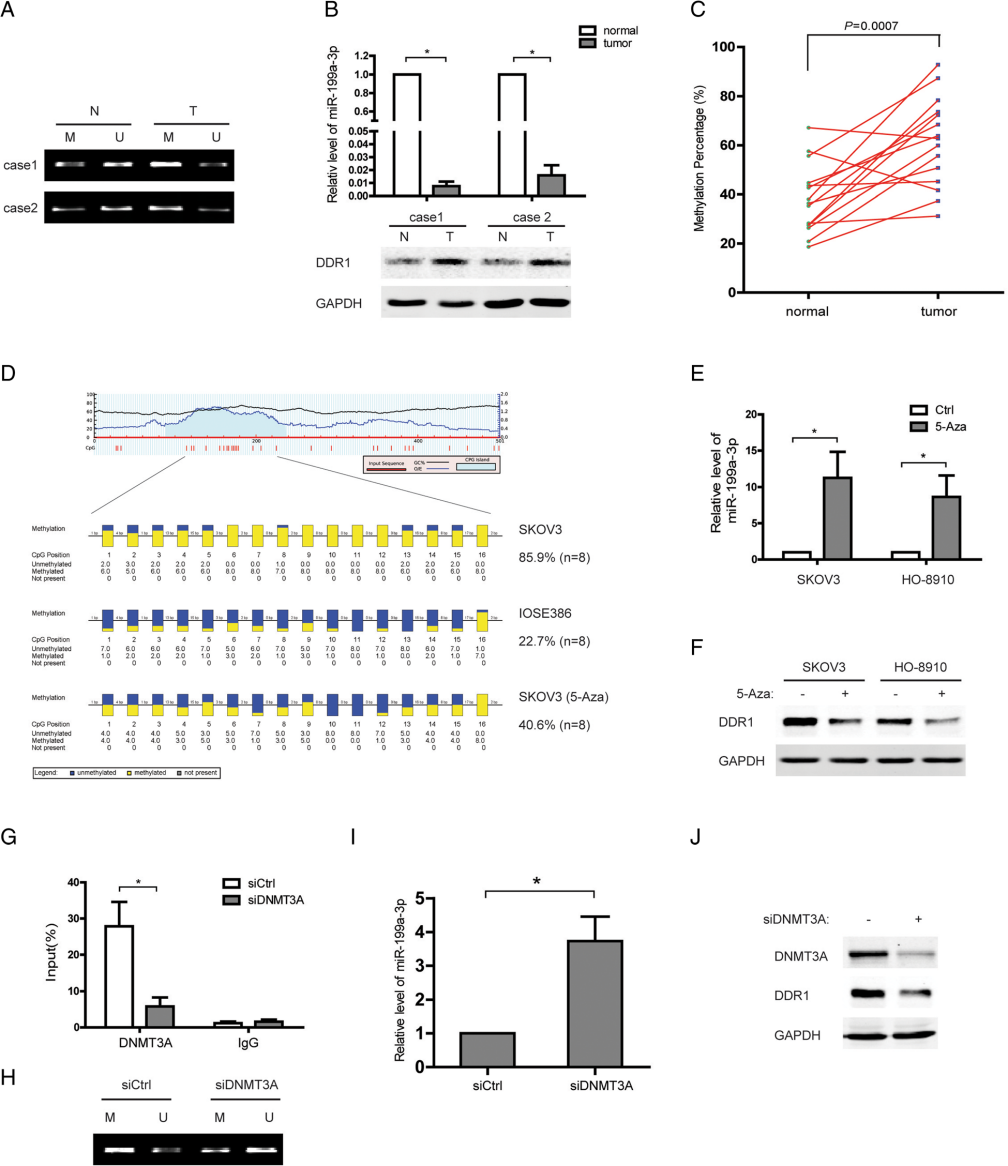
Promoter hypermethylation leads to downregulation of miR-199a-3p in ovarian cancer. **a** Representative agarose gel MSP images of two ovarian cancer cases with paired normal surrounding tissues. **b** miR-199a-3p and DDR1 levels in the two ovarian cancer cases of (**a**) were measured. *P < 0.05. **c** Methylation percentage of miR-199a gene in 15 ovarian cancer cases with paired normal surrounding tissues. The methylation percentage was estimated using the following formula: methylatede(%) = 1/(1 + 2^CtU-CtM^). **d** The hypermethylation levels of miR-199a gene promoters in IOSE386, SKOV3, and 5-Aza-dC treated SKOV3 cells were determined by bisulphite DNA sequencing showing eight independent clones from each group. **e** Relative level of miR-199a-3p in SKOV3 and HO-8910 cells after 5-Aza-dC treatment. *P < 0.05. **f** The protein level of DDR1 in SKOV3 and HO-8910 cells after 5-Aza-dC treatment. **g** SKOV3 cells were transfected with DNMT3A siRNA or control siRNA. ChIP sample was analysed using antibodies against IgG or DNMT3A. The antibody-promoter-binding signals were analysed by quantitative PCR. *P < 0.05. **h-j** SKOV3 cells were transfected with DNMT3A siRNA or control siRNA. The methylation levels of miR-199a gene promoter determined by MSP analyses (**h**), relative levels of miR-199a-3p measured by QPCR (**i**), and the protein levels of DDR1 detected by western blot (**j**). *P < 0.05

As a principal epigenetic modification, DNA hypermethylation has been reported to be associated with aberrant expression profiles of tumor suppressors, especially miRNA. Thus, the methylation status of miR-199a promoter was focused to investigate the mechanisms behind the deregulation of miR-199a-3p in ovarian cancer. Considering that miR-199a is encoded by two loci in the human genome, miR-199a-1 in Chr 19 and miR-199a-2 in Chr 1, the promoters of miR-199a at both loci were analyzed using the software ‘CpGplot’. A region (−404/−272) with high CG composition was detected upstream the transcription start site of miR-199a-1 (Fig. 3d). Therefore, the genome DNA from ovarian cancer cells or tissues was subjected to bisulfate conversion and to a subsequent methylation-specific PCR (MSP) or bisulfite genomic sequencing (BSP). MSP results showed that the methylation levels around the region (−404/−272) were increased in ovarian cancer tissues, compared with adjacent normal tissues (Fig. 3a and c). As expected, the hypermethylation was closely related with decreased miR-199a-3p and upregulation of DDR1 in the ovarian cancer specimens (Fig. 3b). Consistent with above results, high methylation levels of the region (−404/−272) were observed in ovarian cancer cells, SKOV3but not in ovarian epithelial cells, IOSE386 (Fig. 3d). Of note, treatment with a selective inhibitor of DNA methyltransferases (5-Aza-dC) reversed the hypermethylated status in SKOV3 cells (Fig. 3d). In line with this, we found that the expression of miR-199a-3p significantly increased while DDR1 were intensively downregulated after 5-Aza-dC addition, suggesting a hypermethylation mediated loss of miR-199a-3p in ovarian cancer (Fig. 3e and f). Considering that DNMT3A is primarily responsible for the 5mC maintenance, we examined the role of DNMT3A in the methylation of miR-199a promoter in ovarian cancer cells. It was found that DNMT3A knockdown disrupted its binding to the region (−404/−272), the methylation level of which was also decreased (Fig. 3g and h). Furthermore, deprivation of DNMT3A restored the expression of miR-199a-3p and resulted in a reduced DDR1 expression in ovarian cancer cells (Fig. 3i and j). In conclusion, these results suggest that the loss of miR-199-3p expression in ovarian cancer is due to promoter hypermethylation which is enhanced by DNMT3A.

### DDR1 functions as a downstream effector of miR-199a-3p on the migration, invasion, and tumorigenicityof ovarian cancer cells

**Fig. 4:**
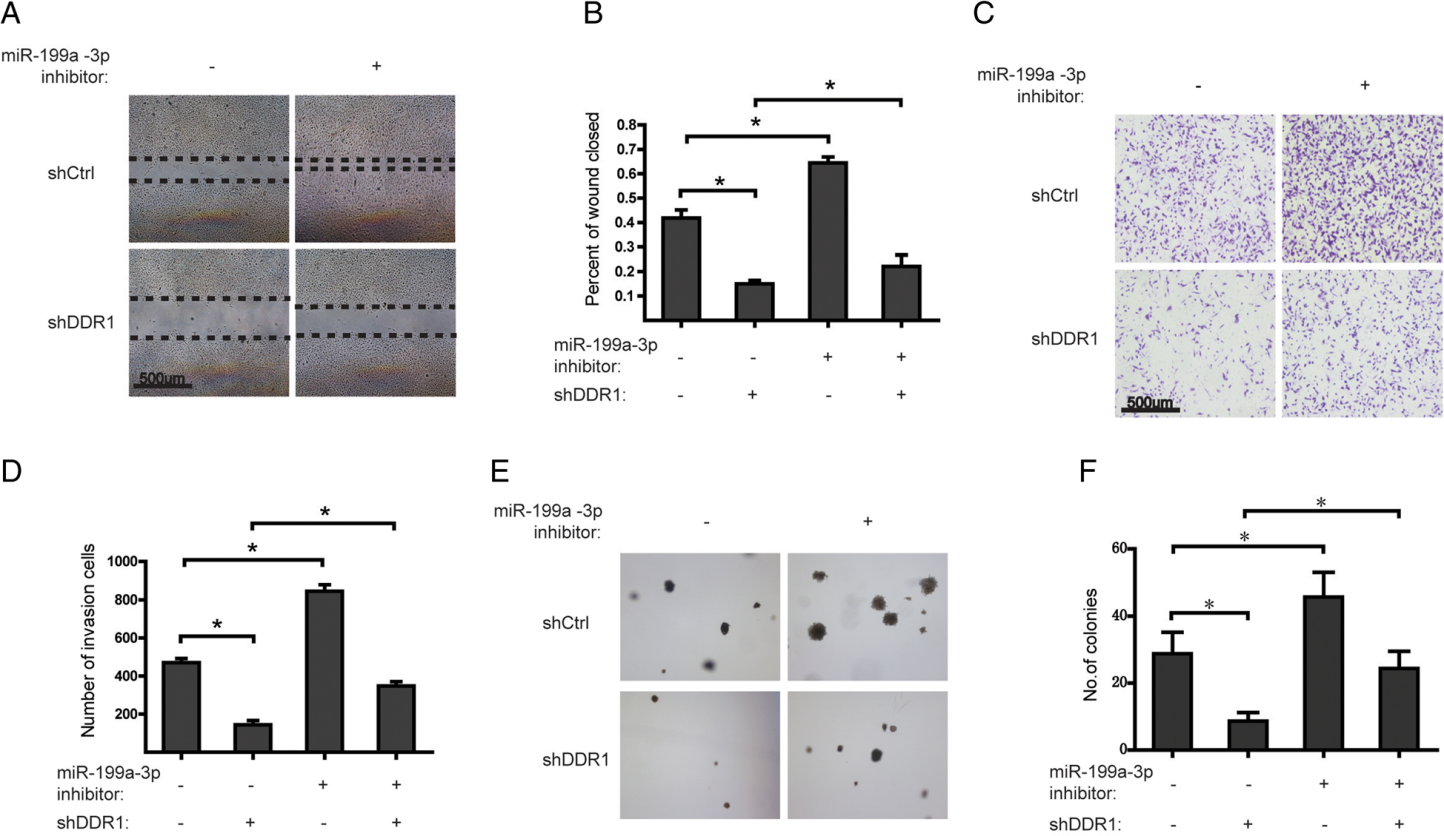
DDR1 functions as a downstream effector of miR-199a-3p on the migration, invasion, and tumorigenicityof ovarian cancer cells. **a** Photographs of the scratch wound assay after stable knockdown of DDR1 or treatment with an anti-miRNA inhibitor specific for miR-199a-3p or negative control in SKOV3 cells. **b** Relative wound closure rates were measured 48 h after indicated treatments for three independent experiments. *P < 0.05. **c** Photographs of the transwell assay after the same treatments as (**a**). **d** The numbers of invasion cells were counted 48 h after indicated treatments for three independent experiments. *P < 0.05. **e** Representative images of colony formation in SKOV3 cells with the same treatments as (**a**). **f** Quantification of colony formation in (**e**). *P < 0.05

The roles of DDRs in cancer progression and metastasis were discussed in different types of tumor. So we want to elucidate whether silencing miR-199a-3p promotes the malignant phenotypes of ovarian cancer via induction of DDR1. The results of wound healing assays showed that SKOV3 cells transfected with miR-199a-3p inhibitor presented a faster closing of scratch wound, compared with the control cells. However, stable knockdown of DDR1 weakened the promotive effect of miR-199a-3p inhibitor on wound healing (Fig. 4a and b). Similarly, cell invasion assays indicated that miR-199a-3p inhibitor resulted in an increased invasion rate of SKOV3 cells compared with the control; while the invasion induced by miR-199a-3p inhibitor was impeded following inhibition of DDR1 (Fig. 4c and d). Consistently, miR-199a-3p inhibitor was found to promote the tumorigenicity of ovarian cancer cells, however, which was disrupted by DDR1 knockdown (Fig. 4e and f). These data indicate that miR-199a-3p may influence the migratory, invasive, and tumorigenic capabilities of ovarian cancer cells via regulating DDR1 expression.

### Dysregulation of miR-199a-3p/DDR1 pathway confers the cisplatin resistance in ovarian cancer

**Fig. 5:**
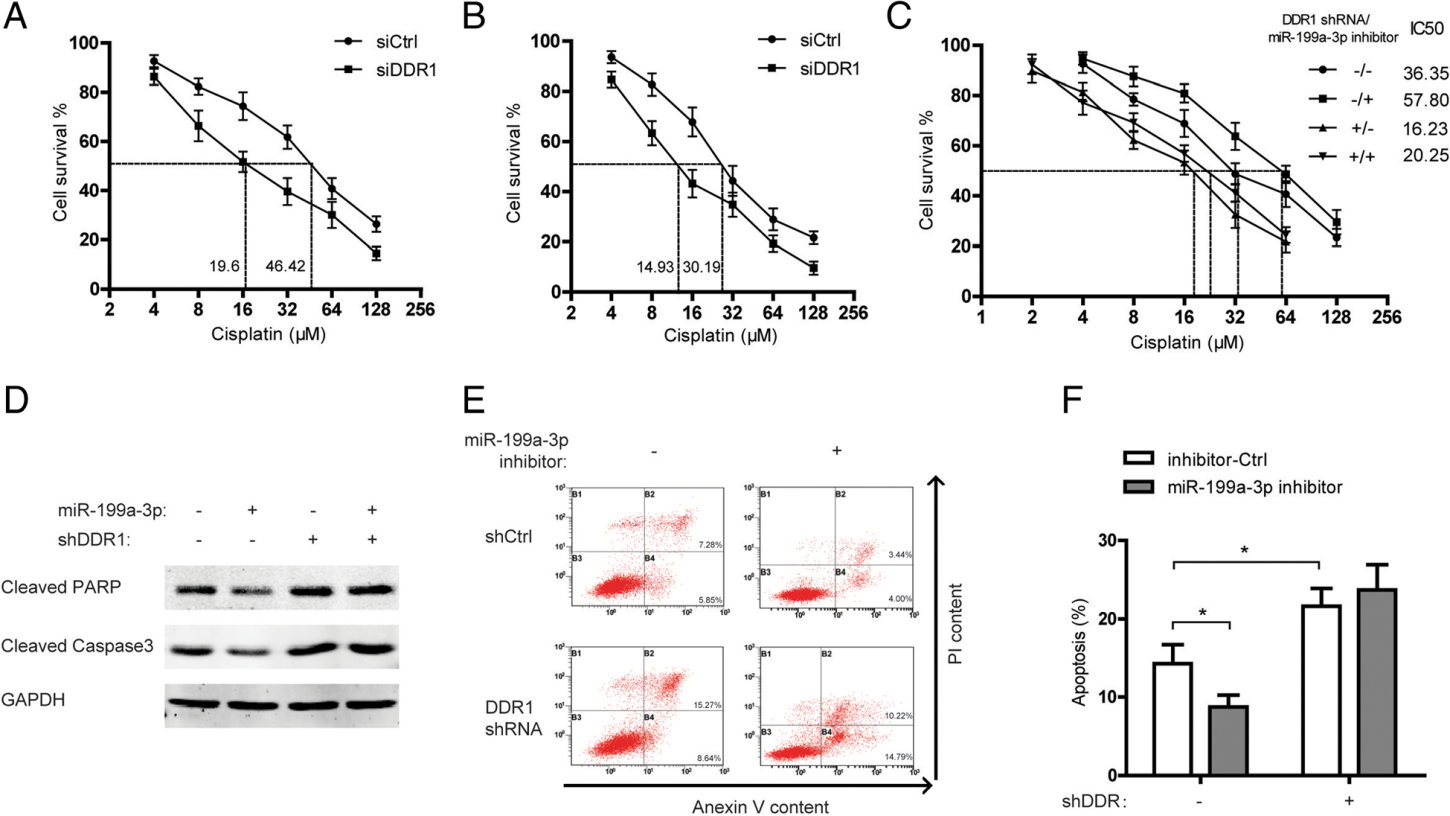
Dysregulation of miR-199a-3p/DDR1 pathway confers the cisplatinresistance in ovarian cancer. **a-c** The viability of SKOV3 cells with knockdown of DDR1 (**a**), HO-8910 cells with knockdown of DDR1 (**b**), and SKOV3 cells with stable knockdown of DDR1 or miR-199a-3p inhibitor treatment (**c**) for 48 h were determined by the CCK-8 assay under different concentrations of cisplatin. **d** Cleaved PARP and caspase 3 in the SKOV3 cells with stable knockdown of DDR1 or miR-199a-3p inhibitor treatment were detected 48 h after 10 μM cisplatin addition. **e** Apoptosis levels of the SKOV3 cells with the same treatments as (**d**) were measured 48 h after 10 μM cisplatin addition. **f** Analysis of three independent experiments in (**e**). *P < 0.05

Dysregulation of miR-199a induced resistance to platinum-based chemotherapy has been widely observed in various cancer types. Therefore, we investigated the involvement of miR-199a-3p and DDR1 in regulating cisplatin sensitivity in ovarian cancer cells. As shown, knockdown of DDR1 by siRNA significantly increased the sensitivity of SKOV3 and HO-8910 cells to cisplatin treatment (Fig. 5a and b). Next, we examined whether knockdown of DDR1was able to override the cisplatin resistance induced by miR-199a-3p inhibitor in ovarian cancer cells. The viability assay revealed that miR-199a-3p inhibitor enhanced the cisplatin resistance of SKOV3 cells without silence of DDR1 but had no evident effect on the viability of SKOV3 cells with stable knockdown of DDR1 following cisplatin treatment (Fig. 5c). Apoptosis assays indicated that miR-199a-3p inhibitor antagonized the cisplatin induced cell apoptosis while elimination of DDR1 by shRNA abrogated the suppressive effect of miR-199a-3p inhibitor on apoptosis (Fig. 5d-f). Taken together, miR-199a-3p/DDR1pathway is crucial for the resistance to cisplatin in ovarian cancer.

## Discussion

In the previous reports, overexpression of DDR1 was shown in ovarian cancer and identified as a potential marker for high-grade ovarian cancer [18, 19]. In the present study, loss of miR-199a-3p was found to cause the ectopic high expression of DDR1 in ovarian cancer. Bioinformation analysis revealed a conserved site at the 3’UTR of DDR1 mRNA, which was targeted by miR-199a-3p. Luciferase reporter assay also confirmed that, as a receptor tyrosine kinase, DDR1 was strictly regulated by the tumor suppressor miRNA, miR-199a-3p. Interestingly, previous findings suggested that miRNA-199a-5p directly regulated the expression of DDR1 in hepatocellular carcinoma [20]. However, unlike the hypermethylation-dependent silence of miR-199a described in the present study, a recent work by Matà et al. demonstrated that IGF-I suppressed miR-199a-5p via activating PI3K/AKT signaling and consequently increased DDR1 expression in breast cancer [21]. Therefore, diverse mechanisms may be employed for regulating miR-199a/DDR1 pathway on the different cellular context. Except for miRNA interference, the studies for regulation of DDR1 expression were limited. During the EMT process of breast epithelial cells, DDR1 was found as a transcriptional target of Zeb1 [22]. Besides, ERK1/2 mediated recruitment of PEA3 to the DDR1 promoter contributed to the DDR1 expression induced by collagen I [23]. Thus, diverse regulatory factors and pathways are involved in controlling DDR1 expression in different pathological context.

DNA hypermethylation, a principal epigenetic modification occurring in CpG islands, has been shown to be associated with aberrant miRNA expression profiles in cancer [24]. Due to that the loci of miR-199a were identified at different chromosomes, the two promoters of miR-199a were firstly analyzed by bioinformation software to detect the potential CpG islands. A CpG-rich region was identified upstream the transcription start site of miR-199a-1 gene located at Chr 19. However, no classical CpG islands were found in the proximal promoter of miR-199a-2 which is located at Chr 1. Therefore, we focused on the methylation status of miR-199a-1 promoter in this study. DNA methylation analysis by MSP indicated a higher methylation level of miR-199a promoter in ovarian cancer, compared with normal tissues. More details of the methylation status at the CpG island were further studied by BSP in ovarian cancer cells. Notably, seven sequential CpGs showed a higher methylation level than others. However, in ovarian epithelial cells, a lower methylation status was observed in the seven sequential CpGs compared with others. In addition, the seven sequential CpGs but not others in the CpG island were strikingly demethylated following 5-Aza-dC treatment in ovarian cancer cells. So, it is reasonable to hypothesize that the seven CpGs may function as a core regulatory element in the CpG island. As a crucial regulator of methylation, DNMT3A mediates the epigenetic silencing of tumor suppressor genes and then contributes to the progression of cancer [25]. Several evidences showed that DNMT3A was highly expressed in various cancers including ovarian carcinoma [26]. In the present work, we found that RNAi mediated depletion of DNMT3A restored miR-199a-3p expression through the reversal of DNA hypermethylation. Interestingly, DNMT3A was identified as another target of miR-199a-3p [27]. Because of promoter hypermethylation, the reduction of miR-199a-3p may induce the upregulation of DNMT3A, which in turn enhances the hypermethylation. Thus, a positive feedback loop may display a critical role in maintaining hypermethylation status and silence of miR-199a-3p in ovarian cancer.

In this study, we found that DDR1 not only functioned as a target of miR-199a-3p but also mediated the malignant phenotypes induced by miR-199a-3p loss. In physiological conditions, DDR-mediated cell migration or adhesion is required in healthy adults. However, in cancer, DDR1 induces tumor metastasis via promoting cell migration and invasion, which is also observed in our study for ovarian cancer. As a signaling mediator, DDR1 regulates diverse downstream effectors which confer the aggressive behaviors of DDR1 in different cancers. In EMT progress, DDR1 was found to interact with α2β1 integrin receptors and activate cell signaling pathways, which increased the expression of mesenchymal markers [28]. In breast cancer, DDR1 accelerated cell migration through blocking the migration suppressor tyrosine-protein kinase (SYK) activity [29]. Accumulating evidences also showed that DDR1 functioned as an inducer of MMP2 and MMP9, which contributed to matrix components degradation during tumor invasion [30–32]. Hence, the downstream factors or pathways of DDR1, which are responsible for ovarian cancer development, are worth investigating in the future.

Overactivation of miR-199a-3p/DDR1 pathway was observed in the clinical specimens of ovarian cancer, as described by highly expressed miR-199a-3p and DDR1 (Fig. 1). However, due to collogen-dependent activation of DDR1, the behaviors of DDR1 may also rely on tumor microenvironment. Therefore, in vivo experiments were widely performed to examine the roles of DDR1 and its related signaling in tumor development [33, 34]. However, based on our results which were limited to in vitro phenotypes, the effects of miR-199a-3p and DDR1 on ovarian cancer metastasis in vivo still remain unverified.

## Conclusions

In summary, our findings highlight a miR-199a-3p/DDR1 pathway, the dysregulation of which leads to the migration, invasion, and chemoresistance of ovarian cancer. Furthermore, our study provides a detailed insight into the mechanism for the miR-199a-3p loss in ovarian cancer. Hypermethylation mediated silence of miR-199a-3p is confirmed to promote DDR1 expression, which is associated with a poor prognosis in patients with ovarian cancer. Therefore, more investigations are needed to explore the potential of miR-199a-3p and DDR1 as novel therapeutic targets for ovarian cancer.

## Abbreviations

DDR1: Discoidin Domain Receptor 1
DNMT3A: DNA methyltransferase 3A
MSP: Methylation-specific PCR
BSP: Bisulfite genomic sequencing
SYK: suppressor tyrosine-protein kinase
